# A general homeostatic principle following lesion induced dendritic remodeling

**DOI:** 10.1186/s40478-016-0285-8

**Published:** 2016-02-25

**Authors:** Steffen Platschek, Hermann Cuntz, Mario Vuksic, Thomas Deller, Peter Jedlicka

**Affiliations:** Institute of Clinical Neuroanatomy, Neuroscience Center, Goethe-University Frankfurt, D-60590 Frankfurt/Main, Germany; Ernst Strüngmann Institute (ESI) for Neuroscience in Cooperation with Max Planck Society, D-60528 Frankfurt/Main, Germany; Frankfurt Institute for Advanced Studies, D-60438 Frankfurt/Main, Germany; Croatian Institute for Brain Research, School of Medicine, University of Zagreb, Salata 12, HR-10000, Zagreb, Croatia

**Keywords:** Electrotonic analysis, Computer simulation, Compartmental modeling, Morphological modeling, Voltage attenuation, Backpropagating action potential, Homeostatic plasticity, Granule cell

## Abstract

**Introduction:**

Neuronal death and subsequent denervation of target areas are hallmarks of many neurological disorders. Denervated neurons lose part of their dendritic tree, and are considered "atrophic", i.e. pathologically altered and damaged. The functional consequences of this phenomenon are poorly understood.

**Results:**

Using computational modelling of 3D-reconstructed granule cells we show that denervation-induced dendritic atrophy also subserves homeostatic functions: By shortening their dendritic tree, granule cells compensate for the loss of inputs by a precise adjustment of excitability. As a consequence, surviving afferents are able to activate the cells, thereby allowing information to flow again through the denervated area. In addition, action potentials backpropagating from the soma to the synapses are enhanced specifically in reorganized portions of the dendritic arbor, resulting in their increased synaptic plasticity. These two observations generalize to any given dendritic tree undergoing structural changes.

**Conclusions:**

Structural homeostatic plasticity, i.e. homeostatic dendritic remodeling, is operating in long-term denervated neurons to achieve functional homeostasis.

**Electronic supplementary material:**

The online version of this article (doi:10.1186/s40478-016-0285-8) contains supplementary material, which is available to authorized users.

## Introduction

Neuronal death is a consequence of a number of neurological disorders such as brain trauma, ischemia or neurodegeneration. However, in contrast to damage to other organs of the body, brain damage is not limited to the damaged site but also affects those areas of the brain which are heavily connected to the site of injury. The target neurons of the injured dying neurons are denervated and show a profound reorganization of their denervated dendritic tree [1, 2]. The mechanisms involved in this postsynaptic reorganization are not yet fully understood. Degeneration of axon terminals may directly affect the postsynaptic target cell by a decrease in afferent drive, loss of neurotrophic factors, and/or loss of adhesion-molecule signaling [1, 3, 4]. Activated micro- and astroglial cells could contribute by changing the composition of the extracellular matrix [5], or the secretion of cyto-[6], and chemokines [7]. Finally, collateral sprouting could contribute to a partial recovery of the dendritic arbor [2, 8–10].

**Fig. 1:**
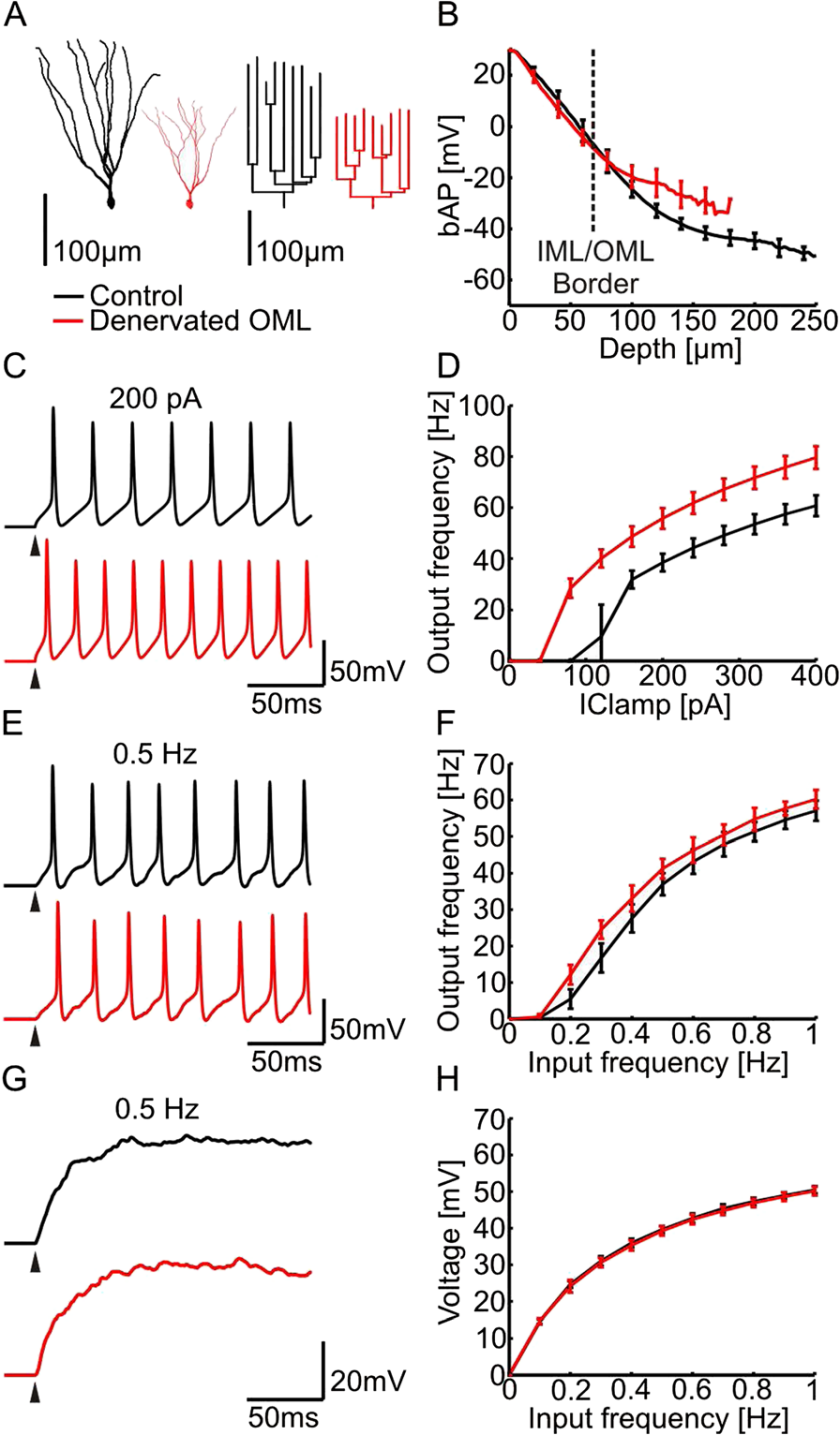
Action potential backpropagation and excitability in compartmental models of denervated granule cell dendrites. **a** Representative examples for 3-D reconstructions of control (*black*) and denervated (*red*) GFP-positive granule cells [10]. Dendrograms of each tree are shown to the right. **b** Simulated action potential backpropagation (bAP) is plotted for control (*black*) and denervated (*red*) granule cells (*n* = 15, each) as a function of depth in the molecular layer. The border between inner and outer molecular layer is indicated by a *dashed line*. The IML/OML layer boundary was obtained from the reconstructed cell data. **c** Somatic membrane voltage traces in active compartmental models of a control (*black*) and a denervated (*red*) granule cell in response to +200 pA somatic current injections. Note an increase in the number of evoked action potentials in the denervated granule cell. **d** Compartmental models of denervated cells displayed higher firing rates following current injections in the soma. **e** Somatic membrane voltage traces in response to stochastic activation of dendritic synapses at 0.5 Hz in the active compartmental model. Excitatory synapses were distributed in dendrites of granule cells using the same synaptic density in both groups. On average 5716 and 3045 synapses were inserted in control and denervated neurons. **f** Corresponding firing rates in response to increasing frequency of stochastic synaptic activation. **g** Somatic membrane voltage traces in response to stochastic activation of dendritic synapses at 0.5 Hz in the passive compartmental model. **h** Corresponding steady-state voltages as a function of frequency of stochastic synaptic activation (same cells as in **b**)

The phenomenon of denervation-induced dendritic remodeling has been studied in some detail using the classical “entorhinal cortex lesion (ECL) model” [2, 3, 11]. In this experimental setting entorhinal afferents to dentate granule cells are lost and granule cells profoundly remodel their dendritic tree [8, 10, 12–14]. Using morphometric analysis of Thy1-GFP mice we recently documented these changes in neuronal arborization in adult mice and showed that entorhinal denervation causes a loss of dendrites resulting in less complex dendritic arbors and a persistent shortening of the dendritic tree [10] (Fig. 1a). The functional consequence of this shortening, which typically has been regarded as “atrophic”, damaging or detrimental to the denervated neuron, has not been studied [4] and remained elusive.

Earlier studies employing computer simulations of neurons have shown that dendritic morphology is a critical determinant of neuronal excitability and firing properties [15–21]. In particular, the electrotonic extent of the dendritic tree appears to have a major impact on neuronal firing [19, 22] as well as the spread, integration and plasticity of synaptic potentials [23–26]. The exceptionally thin dendrites of granule cells are hard to reach with dendritic patch-clamp recordings [27, 28]. We have therefore used experimentally validated anatomically and biophysically realistic compartmental models of 3D-reconstructed healthy and denervated granule cells [10, 29] and have studied the consequences of deafferentation-induced dendritic changes using a comparative electrotonic analysis. We show in the following that dendritic retraction in granule cells and, more generally, in all models of dendritic trees, boosts backpropagating action potentials (bAPs) selectively in the denervated dendritic region. This structural plasticity may serve as a homeostatic mechanism that precisely maintains input-output firing relations.

## Results

### Backpropagating action potentials (bAPs) in denervated granule cell dendrites

By combining full 3D-reconstructions [10] with published values of passive membrane and cytoplasmic parameters [26], we obtained compartmental models for control (healthy) deafferented and denervated dentate gyrus granule cells (see Fig. 1a, Methods). Intuitively, the electrotonic distances within the smaller denervated cells should be shorter. Indeed, in the passive compartmental models, the outward voltage attenuation *L*_*out*_ from the soma toward the dendrites was strongly reduced in denervated granule cells (Additional file 1: Figure S1 and Figure S2). This effect was strongest for high frequency stimulation (40 Hz) and we further quantified the effect by determining mean attenuation distances over the somatodendritic extent of the dendritic tree (see Methods) [30]. We observed significant differences in L_out_ (*p* < 0.0001). Average L_out_ values were 0.15 ± 0.02 (Control) and 0.07 ± 0.02 (Denervated) at 40 Hz stimulation frequency. We conclude from this that the dendritic reorganization after ECL leads to highly significant changes in the electrotonic architecture of denervated granule cells.

The strong reduction in *L*_*out*_ suggests a higher efficacy of voltage spread for somatic action potentials in deafferented neurons relative to control cells. To test this prediction, we simulated backpropagating action potentials (bAPs) by voltage-clamp of a spike shape in the soma of a passive compartmental model [26]. Control cells exhibited realistic attenuation of bAPs along the depth of the molecular layer [28]. In line with the decreased *L*_*out*_, the backpropagation efficacy was higher in denervated than in control granule cells (Fig. 1b). Similar results were obtained when simulating bAPs in active compartmental models [28] (see Methods and Additional file 1: Figure S3E). Most strikingly, significant increase in maximum bAP amplitudes was observed selectively in the denervated dendritic layer (Fig. 1b), i.e. in the outer molecular layer (OML) but not in the inner molecular layer (IML). To delineate the IML, we have adopted the OML/IML border which was identified previously in experimental data as the termination zone of GFP-positive mossy cell axons [10]. On average, peak bAP values in the OML increased from -22.7 ± 3.2 mV to -18 ± 3.9 mV in denervated granule cells as compared to control cells (*p* < 0.001). This result suggests that lesion-induced changes in dendritic morphology facilitate the invasion of bAPs selectively into the deafferented dendritic region.

### Excitability in compartmental models of control and denervated granule cells

Since synaptic events act like current sources rather than voltage sources [24], the spread of synaptic potentials from dendritic locations toward the soma can be predicted by calculating the transfer impedance using high frequency sinusoidal current injections (40 Hz). In our model the transfer impedance revealed significant differences between control and denervated granule cells (*p* < 0.0001; see also Additional file 1: Figure S2), with transfer impedance in denervated cells being larger independently of the distance from the soma. Also the steady-state input impedance was significantly higher in denervated neurons as compared to healthy controls (somatic steady-state input impedance: Control: 349.5 ± 38.8 MΩ vs. Denervated: 591.3 ± 63.2 MΩ; *p* < 0.0001). As predicted from the transfer impedance analysis, explicit simulations of excitatory postsynaptic potential (EPSP) propagation from distal dendritic sites to the soma showed increased amplitudes in denervated granule cells as compared to their control counterparts (*p* < 0.0001). The mean of the average somatic EPSP showed a significant increase from 1.1 ± 0.1 mV to 1.7 ± 0.2 mV. In contrast, the membrane time constant was not significantly changed (Control: 34 ± 0.6 ms vs. Denervated: 34.3 ± 0.4 ms; *p* > 0.1).

To predict the consequences of dendritic atrophy for the granule cell input-output function we added to the passive model active channels for generating realistic spiking [31]. As predicted by the higher input resistances in compartmental models of denervated granule cells, the somatic f-I curves were shifted, rendering the neurons more excitable (Fig. 1c, d; see also Additional file 1: Figure S3). The mean output frequencies in somatic f-I curves (Additional file 1: Figure S3*B*) were strongly increased in denervated cells as compared to control cells. However, when we distributed synapses in dendrites at the same density for compartmental models of both denervated and control cells, we observed similar firing rates (Fig. 1e, f, see also Additional file 1: Figure S3). Thus, in synaptic f-I curves, the greater excitability effectively compensated for the smaller actual number of synapses in the shorter dendrites. Importantly, such remarkable homeostatic compensation for the lower number of synaptic inputs was already present in the passive model, leading to similar somatic voltage output in control and denervated cells when all dendritic synapses, distributed again at the same density, were activated (Fig. 1g, h). Viewed together, these simulations demonstrate that dendritic remodeling following entorhinal denervation enhances the firing ability of dentate gyrus granule cells and contributes to a homeostatic regulation of their synaptically driven output.

### Electrotonic consequences of dendritic remodeling in a morphological model

**Fig. 2:**
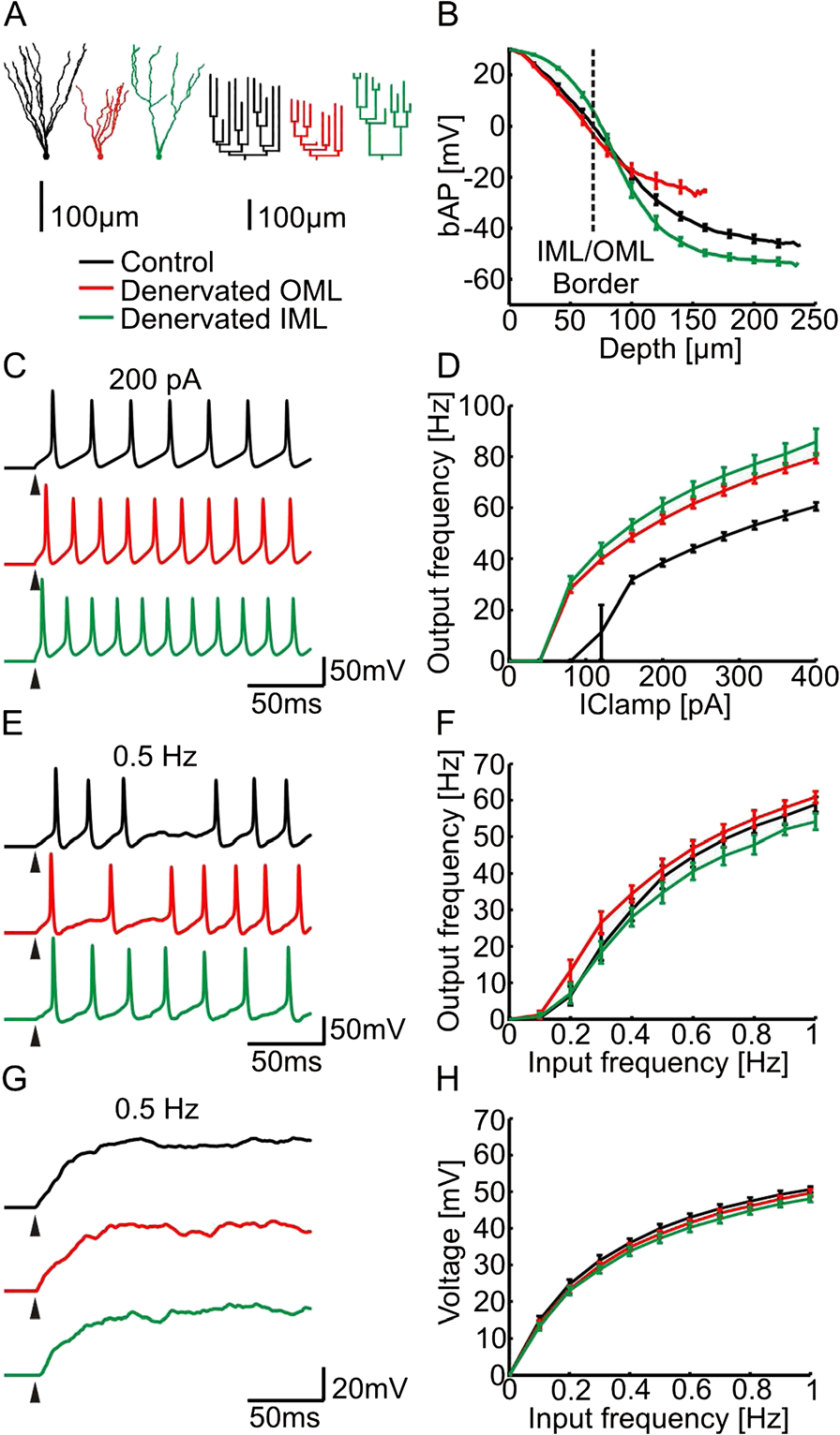
Action potential backpropagation and excitability in compartmental models of granule cells with synthetic morphologies. **a** Representative synthetic morphologies of control (*black*) and denervated granule cells (*red* – denervation in the distal OML; *green* – corresponding to a hypothetical denervation in the proximal IML) including dendrograms. **b**-**f** Action potential backpropagation (bAP), passive and active input-output responses and example voltage traces as in Fig. 1b-h

To better understand the changes in the electrotonic architecture due to lesion-induced dendritic reorganization, we generated synthetic dendritic trees of dentate gyrus granule cells using a morphological model based on optimal wiring principles [32–34]. After target points were positioned in space, the algorithm that was used for the morphological model connected these targets while minimizing total dendrite length and conduction times in the tree. In order to generate synthetic dentate gyrus granule cell morphologies, we positioned target points in the IML and OML to reproduce real granule cell reconstructions and connected these to a tree [34] (Control, Fig. 2a black tree; see Methods). Synthetic denervated cells were reproduced by removing target points in the OML (Denervated OML, Fig. 2a red tree) in a manner to reproduce morphologies of real denervated granule cells (see Methods and Additional file 1: Figure S5). In addition to the morphological models that were constrained by real data, we generated synthetic dendritic trees of granule cells where the IML targets were lesioned (Denervated IML, Fig. 2a green tree) carefully reproducing the dendritic spread and the overall dendritic length of the corresponding OML denervation but in the proximal region of the tree (see Methods and Additional file 1: Figure S5).

Next, we tested electrotonic properties and excitability in synthetic dendrites using the morphological model of control and denervated granule cells as described above. The synthetic morphologies produced very similar results as their real counterparts (Fig. 2). Whereas the membrane time constant remained similar in all three groups (Control: 34.3 ± 0.2 ms, Denervated OML: 34.4 ± 0.2 ms, Denervated IML: 31.5 ± 0.1 ms), *L*_*out*_, backpropagation of action potentials, input resistance, transfer impedance and EPSP attenuation were altered both in the OML lesion and in the IML lesion as compared to the control group (*L*_*out*_ 40 Hz: Control: 0.12 ± 0.01, Denervated OML: 0.06 ± 0.01, Denervated IML: 0.18 ± 0.01; input resistance: Control: 347.8 ± 13.8 MΩ, Denervated OML: 583.3 ± 25.5 MΩ, Denervated IML: 605.6 ± 33.1 MΩ; transfer impedance 40 Hz: Control: 43.3 ± 2.3 MΩ, Denervated OML: 68.3 ± 3.4 MΩ, Denervated IML: 91.8 ± 7 MΩ; somatic EPSP: Control: 1.05 ± 0.05 mV, Denervated OML: 1.64 ± 0.08 mV, Denervated IML: 2.17 ± 0.13 mV). In addition, as in real morphologies, increased excitability of synthetic denervated granule cells (Fig. 2c, d) and higher efficacy of voltage propagation from dendrites to soma led to a homeostatic maintenance of synaptically-driven granule cell firing in active models (Fig. 2e, f). This was also the case for synaptically-evoked somatic voltage changes in the passive case (Fig. 2g, h). Most importantly, as shown previously in the real morphologies, we observed a selective increase in the efficacy of bAPs in the deafferented OML for synthetic neurons (Fig. 2b, compare red and black traces; average bAP values in the OML: Control: -21.3 ± 1.5 mV, Denervated OML: -17.3 ± 2.2 mV). Interestingly, the opposite was true for the fabricated IML lesion, where bAPs were selectively enhanced in the IML as compared to the OML (Fig. 2b, green trace; average bAP values in the IML: Control: 17.3 ± 0.8 mV, Denervated IML: 22.3 ± 0.3 mV). These findings indicate that bAPs are selectively enhanced in the dendritic region where the inputs were lesioned.

### General principle for bAP enhancement

**Fig. 3:**
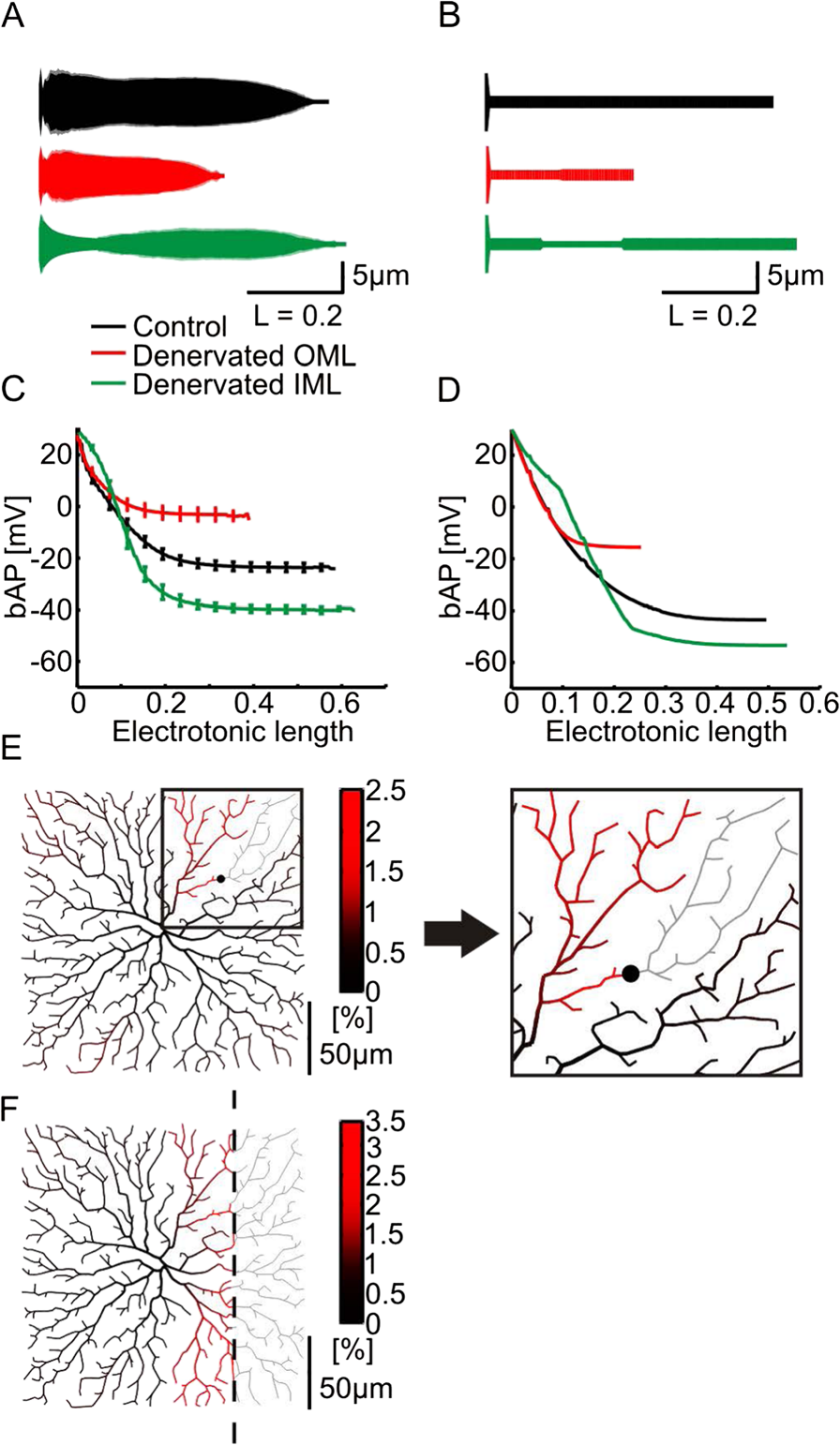
Reduced cable models and morphological models to illustrate the general principle for bAP enhancement. **a** Profiles (radius vs. electrotonic length) of equivalent cable models generated from synthetic cells shown in Fig. 2a (*black* – control; *red* – OML lesion; *green* – IML lesion). The radius of the unbranched equivalent cable was calculated from the radius of dendritic segments at a given electrotonic distance from soma (see Methods). The larger the number of branches at a given electrotonic length, the larger the radius of the equivalent cable. **b** Toy models for all three conditions. **c, d** Simulated action potential backpropagation (bAP) is plotted for the equivalent cables (**c** *, n* = 15, each; see **a**) and toy models (**d**, *n* = 1, each; see **b**) as a function of their electrotonic length. **e** Difference in voltage ratio (%) between lesioned and non-lesioned morphological model grown in a square dendritic field. The lesioned branch is indicated with a *black dot* and *grey dendritic* structure. Inset shows magnified region of the dendrite at which the lesion was made. **f** Similar to **e** but a large region was lesioned. The lesion is indicated with a *dashed line* and *grey dendritic* structure

To understand how bAPs are boosted selectively in the denervated dendrite, we first studied equivalent cable models replicating the electrotonic structure of all three morphological model types (Fig. 3a) [18, 35]. Notably, even the equivalent cable models reproduced the selectively reduced voltage attenuation in the denervated area (Fig. 3a, c). The number of dendritic branch points has been shown previously to be a good predictor of bAP efficacy [18]. The higher the number of branch points the larger the radius of the equivalent cable (see Methods). To further simplify the analysis, we have constructed a reduced version of the equivalent cables (Fig. 3b) by using a simple cylinder with electrotonic distance-dependent changes in the diameter according to equivalent cable parameters. Interestingly, even such reduced models of dendrites were able to replicate bAP amplitudes (Fig. 3d) indicating that structural plasticity very generally underlies compensatory changes in the electrotonic architecture upon denervation.

Exploring dendritic reorganization in a variety of morphological models, we found that the phenomenon that we described for the dentate gyrus granule cell is applicable to any synthetic dendritic tree that we generated. To illustrate this, we demonstrate two conditions: a lesion of a single dendritic branch as well as a lesion of a large dendritic area in a simplified synthetic 2D dendritic tree grown in a square dendritic field. Figure 3e indicates using pseudo-colors the difference between the voltage attenuation (corresponding to action potential backpropagation as seen previously) before and after lesioning a single dendritic branch. Voltage attenuation was selectively reduced in the dendritic area adjacent to the lesion. Selective bAP enhancement in the denervated area was also present when removing a large area of the dendritic tree (Fig. 3f).

### General principle for excitability homeostasis

**Fig. 4:**
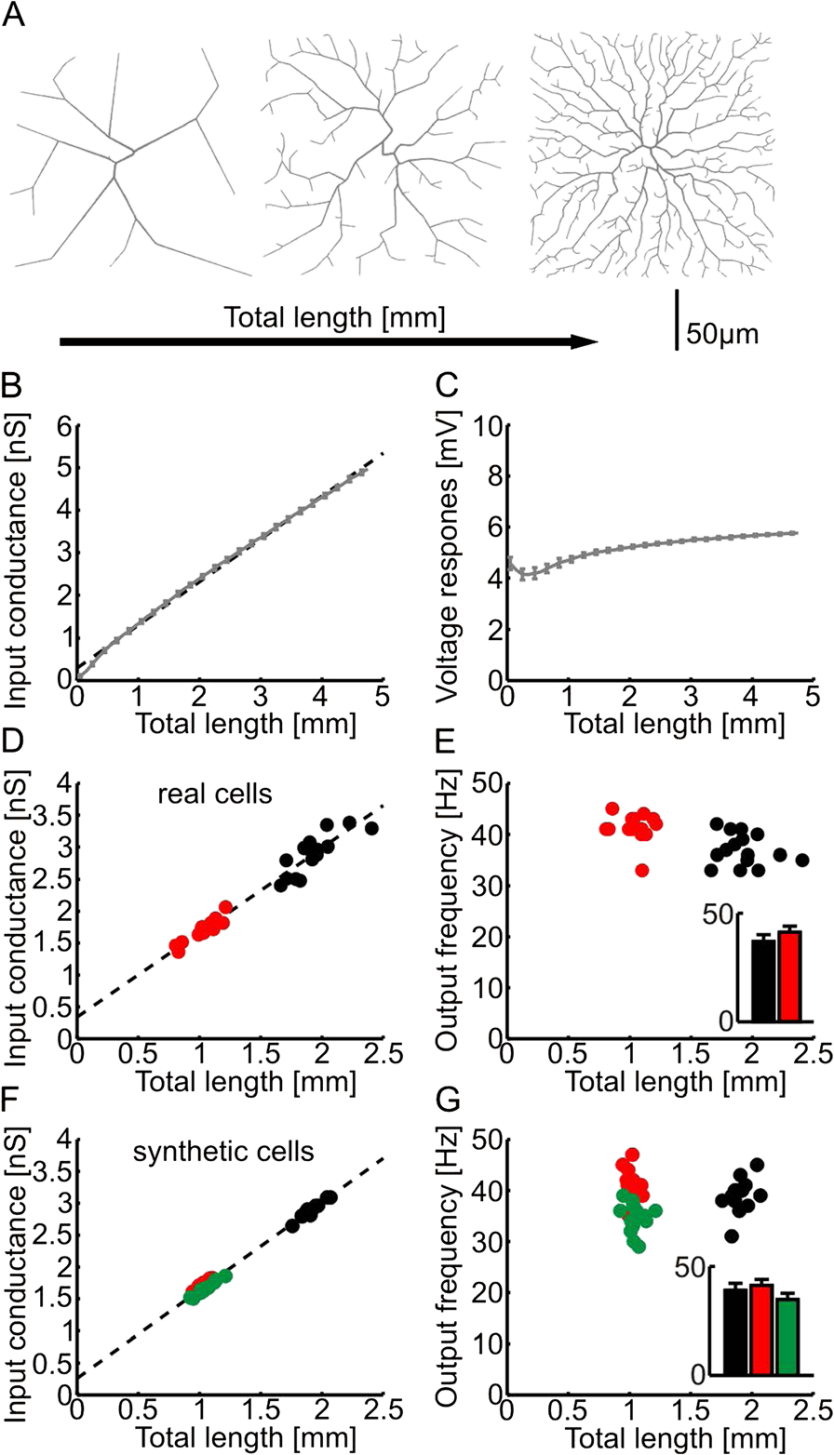
Morphological models reveal the general electrotonic principle leading to the homeostatic regulation of excitability. **a** Representative simplified morphological models (*grey*) with increasing complexity of dendrites. **b** Change of input conductance with total dendrite length in the simplified morphological models (*grey line*) with linear fit (*dashed line*). Single values were averaged in 100 μm sized bins. **c** Resulting somatic membrane potential deflection when activating all synapses, as a function of total dendrite length (*grey line*). An average was performed as in **b**. Synapses were distributed 1 per node and nodes were 1 μm apart. **d**) Input conductance vs. total dendrite length measurements in denervated (*red*) and control *black* granule cell reconstructions with linear fit (*dashed line*). **e** Corresponding firing rate of the cells when all dendritic synapses are activated at 0.5 Hz. The output frequency is plotted versus total dendrite length and summary graph in inset. The same distribution and number of synapses was used as in Fig. 1f. **f** *,* **g** Same as **d** *,* ***e*** but in synthetic cells from Fig. 2. On average 5634, 3032 and 3085 synapses were inserted in synthetic control, denervated OML and denervated IML cells, respectively

The homeostatic regulation of excitability can also be explained using the simple morphological model. We increased the length of synthetic dendrites grown in a square area by increasing the complexity (Fig. 4a). The input conductance increased linearly with the length of dendrite (Fig. 4b). Since the average diameter was the same in all cases and the input conductance increases with membrane surface this is not surprising. Assuming that the synapse density doesn’t change, this means that the number of synapses also grows linearly with the length of dendrite. These two measures cancel each other to reveal an almost constant somatic membrane potential deflection when all synapses are stochastically activated at the same rates (Fig. 4c). For the granule cell synthetic and reconstructed morphologies used in our study the linear relation between total dendrite length and input conductance was true (Fig. 4d and f), which led to an excitability of the cells that was independent of total length (Fig. 4e and g).

## Discussion and conclusions

In this study, we used computational models to predict the effects of dendritic reorganization on the electrotonic structure and intrinsic excitability of deafferented dentate granule cells. In the specific case of the denervated granule cells, our simulations revealed that these neurons are, as expected, electrotonically more compact than control granule cells. As a consequence, backpropagating action potential (bAP) attenuation and dendrosomatic EPSP attenuation were significantly reduced in their denervated dendrites. Notably, this boost of bAPs was restricted to the denervated dendritic layers. Since bAPs that reach back to the synapse are thought to be involved with strengthening synaptic weights locally, this result indicates that dendritic remodeling could contribute to a homeostatic strengthening of surviving synapses on denervated dendritic segments [29, 36]. Moreover, simulations of somatic and dendritic f-I curves revealed an increased excitability of the cells. In conjunction with the smaller number of synapses this led to a homeostatic maintenance of firing rates in denervated granule cells. Thus, the shortening of dendrites helps to restore the normal firing pattern of granule cells and normalizes information throughput to the hippocampus.

Using both simplified passive cable models as well as generalized morphological models [18, 34, 37] we unraveled the general principles that led to these two homeostatic features of dendritic remodeling. We showed that the observed effects are emerging properties of alterations in dendritic electrotonic architecture. Our data imply that denervation-induced structural adaptations of neurons counteract the loss of synaptic inputs due to denervation and thus contribute to neuronal homeostasis. In contrast to the current view, which regards denervation-induced dendritic retraction as detrimental for a neuron, we here propose that this form of dendritic remodeling returns a denervated neuron to its functional state and may, in fact, be restorative.

### Computational models reveal a selective boost of bAPs following dendritic atrophy and retraction

A large number of predictions from passive electrotonic analyses [38–40] have revealed general principles of dendritic computation that were consequently also shown to be true in experiments [41, 42] or complex active computational models [43–45]. Using simplified branched morphological models and the resulting derived compartmental models, we showed that dendritic retraction in passive dendrites leads to a selective boost of bAPs specifically in the retracted dendritic region. This is in line with a previous study that proposed the number of branch points as a predictor of bAP efficacy [18] since reducing the number of dendritic branches decreases the number of branch points [46]. Because of its general applicability, we would like to claim that the principle that we describe in this manuscript will apply under a wide variety of biologically relevant settings in adult animals.

First, we have tested this in the case of the dentate gyrus granule cell. Retracting distal dendrites as a consequence of entorhinal cortex lesion intuitively impinges on (1) the attenuation of electrical signals because of the shorter dendrites and (2) the excitability of the cells because of the higher input resistance. We showed that voltage attenuation in both directions (*L*_*in*_ and *L*_*out*_) was decreased upon denervation independently of the frequency. Interestingly however, fast events were facilitated most strongly in the somatofugal direction favoring action potential backpropagation vs. EPSP forward propagation. The preexisting asymmetric attenuation (*L*_*in*_ > *L*_*out*_) due to open-end versus sealed-end boundary conditions for *L*_*in*_ versus *L*_*out*_ [30, 37, 47–49] was thereby counterbalanced after dendritic retraction. In line with the diminished *L*_*out*_, explicit simulations confirmed the predicted boost of bAPs in dendrites of denervated granule cells. bAPs being a major factor affecting spike-timing dependent long-term synaptic changes [50–53], such synaptic changes would be enhanced specifically where branches have retracted, i.e. at 100–200 μm depth in the molecular layer in the case of the denervated granule cell. The predictions of our model should be tested directly using electrophysiological recordings [28] or calcium/voltage imaging in the dendrites of granule cells. Importantly, the increase in bAPs exclusively in denervated dendritic segments of granule cells could result in the strengthening of surviving excitatory synapses. Such a mechanism could contribute and/or maintain the observed layer-specific homeostatic strengthening of surviving synapses after denervation which we described recently [29, 36]. Furthermore, enhanced bAPs are likely to increase the plasticity of denervated dendritic segments and could thus facilitate the stabilization of new synapses and the rewiring of the denervated segments. All of these effects would counteract the denervation effects and thus appear to be homeostatic in nature.

Second, using a general morphological model, we were able to show that the spatially selective enhancement of action potential backpropagation is not only present in the specific case of denervated granule cells but, remarkably, in any dendritic morphology. The retraction of a single dendritic branch or a large dendritic region will lead to a tightly focused reduction in the attenuation of bAPs in the targeted area. This mechanism will enable any dendritic tree which undergoes structural remodeling including extension or shortening of its distal branches to adjust local synaptic plasticity specifically in the remodeled dendritic subcompartments.

### Precise adjustment of excitability associated with dendritic retraction/atrophy counteracts the effects of denervation

We wondered how functional changes associated with the loss of distal dendritic segments will affect the denervated neurons. Again, we looked first at the specific case of the dentate granule cells, which have been thoroughly investigated following denervation. After denervation the firing rate of these cells decreases and then recovers to control levels by one week post lesion [54]. This indicates that structural and functional changes occur after denervation which restore the excitatory drive of these neurons and thus information throughput via the dentate. Sprouting of surviving fibers and compensatory synaptic scaling were discussed as the major mechanisms contributing to this early restoration of neuronal firing [36, 55]. Our data suggest that, in the next weeks following the denervation, dendritic atrophy could also play an important role in this context by maintaining the firing rates after synaptic scaling has declined. We noted that dendritic atrophy will result in an increased granule cell excitability because of enhanced passive dendrosomatic signal propagation and larger input resistance to somatic and dendritic current injections [20, 22, 56]. This increase in excitability does not, however, result in hippocampal seizures, since the increased excitability of these cells goes hand in hand with a reduced excitatory input. To understand the net effect of this increased excitability we reproduced the circuit input onto denervated granule cells after ECL. Since spine densities of denervated dendritic segments return to normal within the first weeks post-lesion [8, 10, 12] we modeled a similar density of inputs as in control cells [10]. This resulted in a decrease in the absolute number of synapses. Remarkably, the increase in excitability of granule cells after lesion compensated exactly for the resulting smaller number of excitatory synapses, suggesting that these structural changes of the granule cell dendritic tree return granule cell excitability to a physiological working level. In other words, by remodeling the size of its dendritic tree a denervated neuron can exquisitely calibrate its excitability and adapt it to the available afferent input. Such a mechanism counteracts the denervation effects and appears to be a homeostatic mechanism by which neurons regulate their activity under injury conditions. Similarly to the bAP enhancement, using a general morphological model, we have shown that this homeostatic mechanism is not only present in granule cells but represents a fundamental feature of all dendritic trees which undergo lengthening or shortening of their branches while keeping synaptic density constant. A change in input conductance gets canceled out by a change in the number of synapses leading to firing rate independent of the length of dendrites.

### Dendritic atrophy–good or bad for a denervated neuron?

The biological phenomenon of denervation-induced plasticity has been receiving more and more attention [55, 57–60]. It is now well-recognized that the structural reorganization of neurons can contribute to disease pathogenesis [61, 62] as well as to functional regeneration following a partial injury [63, 64]. Dendritic retraction and remodeling are widely seen in neurological diseases and are usually considered signs of pathology and malfunction. In fact, some authors have even linked progressive impairment of dendrite maintenance to cognitive defects [65]. Although we do not dispute that dendritic atrophy is a sign of an abnormal condition, we would like to point out that the long-term functional consequences of limited dendritic atrophy are in part homeostatic and could thus help a neuron to maintain the function of its remaining synapses. Too much dendritic atrophy, however, may exceed the ability of the system to compensate [62]. This ability to compensate may also depend on the age of the neuron, since immature neurons are considerably more and aged neurons are considerably less plastic than the adult neurons on which our study is based [66–68]. Thus, limited dendritic atrophy may be good for the remaining functional activity of a denervated neuron in the network while it may be detrimental to a neuron, or even lethal, if it exceeds a certain neuron or age-specific threshold.

## Methods

### Morphology of reconstructed granule cells

We used detailed 3D reconstructions [10] obtained from high resolution confocal images of GFP-labeled control (*n* = 15) and and long-term denervated (*n* = 15; 90 days after entorhinal cortex lesion) dentate granule cells in sections of Thy1-GFP transgenic mice. Of note, using age-matched controls, the previous experimental study excluded that dendritic retraction may have been induced by aging instead of denervation [10]. The reconstructed neurons were imported into Matlab (Mathworks, Natick, MA) using the TREES toolbox software package [34, 69] (www.treestoolbox.org). The reconstructions were used to directly generate synthetic granule cell morphologies for compartmental modeling (see below). To correct diameter overestimation due to the fluorescence halo in confocal images, a quadratic diameter taper [32] was mapped onto all dendritic tree structures (function “quaddiameter_tree” in the TREES toolbox, see Additional file 1: Figure S4). The tapering was not required for firing rate homeostasis. Reconstruction data from biocytin labeled granule cells [26] were used to constrain the quadratic equation parameters. Additionally, proximal dendrites with a path length smaller than 110 μm were adjusted to match the diameter of the reconstructed granule cells. An axon from Ref. [26] was attached to all morphologies. The digitized morphologies were then exported to NEURON.

### Electrotonic analysis

Electrotonic analyses were carried out with the NEURON simulation program [70]. Passive properties were taken from Schmidt-Hieber et al. [26]: *R*_*a*_ (specific axial resistance) = 194 Ωcm; *R*_*m*_ (specific membrane resistance) = 38 kΩcm^2^; *C*_*m*_ (specific membrane capacitance) = 1.01 μFcm^−2^. Spines were implicitly modeled by scaling *R*_*m*_ and *C*_*m*_ of dendrites to include the spine membrane surface area [31]. Implementing these passive parameters in our reconstructed morphologies reproduced experimental values for membrane time constant and input resistance taken from electrophysiological recordings in typical control granule cells [26]. The passive parameters were kept identical in both control and denervated cells to isolate the unique effects of morphological changes [30]. Membrane time constant was unaltered in denervated cells but input resistance was higher in denervated cells (see Results). Electrotonic lengths (*L*_*in*,_*L*_*out*_) were computed by determining the natural logarithm of the somatopetal and somatofugal voltage attenuation, respectively [30]. Somatofugal (*L*_*out*_) and somatopetal (*L*_*in*_) attenuation were defined by the following function: ${L}_{in}(X)=\frac{{\displaystyle {\sum}_i^n\mathrm{In}\;\left({V}_{remote}{(X)}_i/{V}_{soma}{(X)}_i\right)}}{n}$ and ${L}_{out}(X)=\frac{{\displaystyle {\sum}_i^n\mathrm{In}\;\left({V}_{soma}{(X)}_i/{V}_{remote}{(X)}_i\right)}}{n}$, where *i* runs through all *n* segments of the dendritic tree at a certain somatodendritic depth *X*. The voltage clamp was inserted either in the soma (*L*_*out*_) or in the corresponding segments (*L*_*in*_).

### Simulations of backpropagating action potentials (bAPs)

Passive propagation was simulated as in Refs [18, 30]: a somatic action potential waveform acquired from an active model [71] was injected as a voltage-clamp command at the soma. This was done to isolate action potential propagation from action potential initiation and thus to make sure backpropagation starts from the same initial conditions in each morphology [18]. To study bAPs in the active model, we used a previously published model of a hippocampal dentate gyrus granule cell with biophysical properties tuned to reproduce dendritic recordings of bAPs [28]. Action potentials were initiated by supra-threshold somatic current injection. Similar to the electrotonic analysis, the backpropagation efficacy was quantified by computing the average maximum bAP amplitude across all branches at a given somatodendritic depth: $V(X)=\frac{{\displaystyle {\sum}_i^nV{(X)}_i}}{n}$, where *i* runs through all *n* segments of the dendritic tree at a certain somatodendritic depth *X*.

### Calculation of transfer impedance and simulations of excitatory postsynaptic potential (EPSP) attenuation

Transfer impedance, the ratio of remote voltage change and local current injection, was computed for each segment of all dendritic trees using the impedance tools built into NEURON. These measures included the somatic input resistance where steady state current injection and voltage recording coincide at the soma. Excitatory postsynaptic potentials (EPSPs) were simulated using a bi-exponential synaptic conductance (rise time = 0.2 ms, decay time = 2.5 ms, reversal potential = 0 mV, peak conductance = 1 nS); values from Ref. [26]. Dendrosomatic attenuation of EPSPs was determined by measuring somatic voltage changes in response to excitatory postsynaptic events evoked at all locations of the dendritic tree $EPSP(X)=\frac{{\displaystyle {\sum}_i^n EPSP{(X)}_i}}{n \bullet EPS{P}_{\max }}$, where *i* runs through all *n* segments of the dendritic tree at a certain somatodendritic depth *X*.

### Simulations of somatic and synaptic input-output responses

To determine synaptic input-output relationships, passive parameters were obtained from published data by Schmidt-Hieber et al. [26]. For active simulations we employed two active models of hippocampal dentate gyrus granule cells with realistic biophysical properties previously published by Aradi and Holmes (AH-model) [71–73] and by Schmidt-Hieber et al. (SH-model) [31]. Simulation files were downloaded from the ModelDB database [74] at http://senselab.med.yale.edu/modeldb/. Passive properties were taken from Schmidt-Hieber et al. [26] to generate a passive structure for the insertion of the various active channels. AH-model granule cells comprised five distinct sections with different densities of nine voltage-activated channels: soma, granule cell layer dendritic section, proximal, middle, and distal dendritic section. In the other active model, voltage-dependent Na^+^ and K^+^ channels were inserted into both soma and dendrites. Various f-I relationships are shown in Additional file 1: Figure S3. In order to most accurately estimate the input-output response in denervated granule cells we designed a realistic synaptic input scenario. ECL spine density recovers within 2 weeks after denervation [10], we therefore inserted synapses with the same density (3 ± 0.2 synapses/μm) throughout the whole dendritic tree. The spatial distribution of synapses was homogenous. Synaptic inputs were simulated as conductances with bi-exponential time course with rising and decay time constants of 0.2 and 2.5 ms respectively and with a maximum value of 0.5 nS. Individual synapses were activated by independent (asynchronous) presynaptic spikes. Presynaptic stimulation followed a Poisson distribution [75].

### Statistical analysis

Data are presented as mean ± standard deviation. Statistical comparisons were made using the Wilcoxon–Mann–Whitney test or Kruskal–Wallis test followed by Dunn’s multiple comparison test. A two-tailed p value lower than 0.05 was considered to be significant. Only the layers containing dendrites of all control and denervated cells (*n* = 15 each) were used for the statistical analysis of distance-dependent properties. Therefore, the first 180 μm of the somatodendritic depth of reconstructed trees were used to quantify *L*_*out*_, bAPs, transfer impedance and EPSPs in control and denervated granule cells.

### Morphological models

Synthetic control and denervated granule cell dendrite morphologies were generated connecting targets using a minimum spanning tree algorithm based on wiring optimization principles as described previously [32, 34]. Three different groups each consisting of 15 artificial granule cells were constructed: (1) control granule cells, (2) denervated granule cells which lost their afferent axons in the OML (denervated OML) and (3) hypothetical, denervated granule cells, which lost their afferent axons in the IML (denervated IML). The original control and denervated cell reconstructions were centered on the soma location, rotated and scaled to obtain dendritic fields [34]. Cone shaped dendrite spanning fields for each cell group were defined approximating the dendritic fields and subdivided into volumes with a height of 20 μm. The density profile of topological points (branch and termination points) was obtained from reconstructed dendrites and used to provide target points for the minimum spanning tree algorithm. Target points were randomly distributed within the previously described volumes of the dendrite spanning fields and connected to dendritic structures satisfying two wiring costs: (1) the total amount of wiring should be minimal and (2) the path length from any target point to the soma should be minimal. These two constraints were applied using a balancing factor of 0.98, which weighted the second cost against the first cost. One out of 150 synthetic cells was selected that matched the statistical data best. This resulted in highly realistic values for the number of Sholl-like intersections (Additional file 1: Figure S5A) and the distribution of topological points (Additional file 1: Figure S5B) in the synthetic cells. An axon from a reconstructed granule cell and a realistic soma matching the average length and surface of the somata from reconstructed control and denervated granule cells respectively were added. The IML/OML layer boundary was transferred from the reconstructed cell data. The synthetic cells were refined using a set of functions from the TREES toolbox: resample_tree (distance = 2 μm), smooth_tree (pwchild = 0.5, p = 0.8, n = 10), jitter_tree: parameters were chosen to fit the total dendritic length in each dendritic layer (Additional file 1: Figure S5*C*), quaddiameter_tree (slope = 0.016, offset = 0.65) where proximal dendrites with a path length smaller than 110 μm were adjusted to match the diameter of the reconstructed granule cells. The same active and passive properties were used as in reconstructed cells. To obtain synthetic denervated IML granule cells, a similar approach was pursued. Topology points from reconstructed, denervated OML granule cells were shifted distally by 80 μm, then translated into target points and distributed in the previously described cone shaped dendrite spanning field of artificial, control granule cells. The remaining steps correspond to the generating of synthetic OML denervated granule cells. A set of simplified morphological models were generated by connecting 1 to 1000 targets in a 200 x 200 μm square area with a balancing factor of 0.5 (Fig. 3e, f and Fig. 4a-c). Diameter values were mapped using the quaddiameter_tree function of the TREES toolbox, with a slope of 0.1 and a terminal diameter of 0.5 μm. Passive electrotonic simulations were performed with specific membrane resistance of 20,000 kΩ cm^2^ and axial resistance of 200 Ωcm.

### Equivalent cable models

Equivalent cable models were generated using the TREES toolbox. The radius of the equivalent cables was determined as ${r}_{eq}(X)=\left({\displaystyle {\sum}_ir{(X)}_i^{\frac{3}{2}}}\right){\scriptscriptstyle \frac{2}{3}}$, where *i* is an index of each segment in the synthetic dendritic tree located at an electronic distance *X* from the soma [18].

### Simplified cylinder models

In order to understand the lesion-induced electrotonic changes at the level of cable models we generated simplified cylinder models for control and denervated granule cells. A control case cylinder model consisted of a 10 μm long cylinder of 5 μm radius appended with a 490 μm long cylinder of 1 μm radius. We generated an OML lesion cylinder model by shortening the control model to half of its length and by reducing the proximal radius to 0.83 μm. We generated an IML lesion cylinder model by reducing the radius of the control cylinder to half of its initial value between 20 % and 30 % of its length. Passive properties were taken from Schmidt-Hieber et al. 2007 [26].

## Additional file

Additional file 1: **Supplementary Material.** (PDF 580 kb)

## Abbreviations

bAP: backpropagating action potential
ECL: entorhinal cortex lesion
EPSP: excitatory postsynaptic potential
f-I: frequency-current curve
*L*_*in*_, *L*_*out*_: electrotonic inward and outward attenuation distance
OML, IML: outer and inner molecular layer

## References

[1] Deller T, Del Turco D, Rappert A, Bechmann I (2007). Structural reorganization of the dentate gyrus following entorhinal denervation: species differences between rat and mouse. Prog Brain Res.

[2] Steward O, Salzman SK, Faden AI (1994). Reorganization of neuronal circuitry following central nervous system trauma: naturally occurring processes and opportunities for therapeutic intervention. Neurobiol. Cent. Nerv. Syst.

[3] Deller T, Frotscher M (1997). Lesion-induced plasticity of central neurons: sprouting of single fibres in the rat hippocampus after unilateral entorhinal cortex lesion. Prog Neurobiol.

[4] Perederiy JV, Westbrook GL, Frotscher M, Ludwigs A (2013). Structural plasticity in the dentate gyrus- revisiting a classic injury model. Front Neural Circuits.

[5] Deller T, Haas CA, Frotscher M (2000). Reorganization of the rat fascia dentata after a unilateral entorhinal cortex lesion. Role of the extracellular matrix. Ann N Y Acad Sci.

[6] Becker D, Deller T, Vlachos A (2015). Tumor necrosis factor (TNF)-receptor 1 and 2 mediate homeostatic synaptic plasticity of denervated mouse dentate granule cells. Sci Rep.

[7] Rappert A, Bechmann I, Pivneva T, Mahlo J, Biber K, Nolte C, Kovac AD, Gerard C, Boddeke HWGM, Nitsch R, Kettenmann H (2004). CXCR3-Dependent Microglial Recruitment Is Essential for Dendrite Loss after Brain Lesion. J Neurosci.

[8] Caceres A, Steward O (1983). Dendritic reorganization in the denervated dentate gyrus of the rat following entorhinal cortical lesions: A Golgi and electron microscopic analysis. J Comp Neurol.

[9] Nitsch R, Frotscher M (1991). Maintenance of peripheral dendrites of GABAergic neurons requires specific input. Brain Res.

[10] Vuksic M, Del Turco D, Vlachos A, Schuldt G, Müller CM, Schneider G, Deller T (2011). Unilateral entorhinal denervation leads to long-lasting dendritic alterations of mouse hippocampal granule cells. Exp Neurol.

[11] Cotman CW, Nadler JV, Cotman CW (1978). Reactive synaptogenesis in the hippocampus. Neuronal Plast.

[12] Parnavelas JG, Lynch G, Brecha N, Cotman CW, Globus A (1974). Spine loss and regrowth in hippocampus following deafferentation. Nature.

[13] Schauwecker PE, McNeill TH (1996). Dendritic remodeling of dentate granule cells following a combined entorhinal cortex/fimbria fornix lesion. Exp Neurol.

[14] Diekmann S, Ohm TG, Nitsch R (1996). Long-lasting transneuronal changes in rat dentate granule cell dendrites after entorhinal cortex lesion. A combined intracellular injection and electron microscopy study. Brain Pathol.

[15] Mainen ZF, Sejnowski TJ (1996). Influence of dendritic structure on firing pattern in model neocortical neurons. Nature.

[16] Sheasby BW, Fohlmeister JF (1999). Impulse Encoding Across the Dendritic Morphologies of Retinal Ganglion Cells. J Neurophysiol.

[17] Van Ooyen A, Duijnhouwer J, Remme MWH, Van Pelt J (2002). The effect of dendritic topology on firing patterns in model neurons. Netw Comput Neural Syst.

[18] Vetter P, Roth A, Häusser M (2001). Propagation of action potentials in dendrites depends on dendritic morphology. J Neurophysiol.

[19] Van Elburg RAJ, Van Ooyen A (2010). Impact of Dendritic Size and Dendritic Topology on Burst Firing in Pyramidal Cells. PLoS Comput Biol.

[20] Weaver CM, Wearne SL (2008). Neuronal firing sensitivity to morphologic and active membrane parameters. PLoS Comput Biol.

[21] Krichmar JL, Nasuto SJ, Scorcioni R, Washington SD, Ascoli GA (2002). Effects of dendritic morphology on CA3 pyramidal cell electrophysiology: a simulation study. Brain Res.

[22] Bekkers JM, Häusser M (2007). Targeted dendrotomy reveals active and passive contributions of the dendritic tree to synaptic integration and neuronal output. PNAS.

[23] Rall W, Koch C, Segev I (1989). Cable theory for dendritic neurons. Methods neuronal Model.

[24] Jaffe DB, Carnevale NT (1999). Passive normalization of synaptic integration influenced by dendritic architecture. J Neurophysiol.

[25] Golding NL, Mickus TJ, Katz Y, Kath WL, Spruston N (2005). Factors mediating powerful voltage attenuation along CA1 pyramidal neuron dendrites. J Physiol.

[26] Schmidt-Hieber C, Jonas P, Bischofberger J (2007). Subthreshold dendritic signal processing and coincidence detection in dentate gyrus granule cells. J Neurosci.

[27] Schmidt-Hieber C, Jonas P, Bischofberger J (2008). Action potential initiation and propagation in hippocampal mossy fibre axons. J Physiol.

[28] Krueppel R, Remy S, Beck H (2011). Dendritic integration in hippocampal dentate granule cells. Neuron.

[29] Vlachos A, Becker D, Jedlicka P, Winkels R, Roeper J, Deller T (2012). Entorhinal denervation induces homeostatic synaptic scaling of excitatory postsynapses of dentate granule cells in mouse organotypic slice cultures. PLoS One.

[30] Kabaso D, Coskren PJ, Henry BI, Hof PR, Wearne SL (2009). The electrotonic structure of pyramidal neurons contributing to prefrontal cortical circuits in macaque monkeys is significantly altered in aging. Cereb Cortex.

[31] Schmidt-Hieber C, Bischofberger J (2010). Fast sodium channel gating supports localized and efficient axonal action potential initiation. J Neurosci.

[32] Cuntz H, Borst A, Segev I (2007). Optimization principles of dendritic structure. Theor Biol Med Model.

[33] Cuntz H, Forstner F, Haag J, Borst A (2008). The morphological identity of insect dendrites. PLoS Comput Biol.

[34] Cuntz H, Forstner F, Borst A, Häusser M (2010). One rule to grow them all: a general theory of neuronal branching and its practical application. PLoS Comput Biol.

[35] Rall W, Burke RE, Holmes WR, Jack JJB, Redman SJ, Segev I (1992). Matching dendritic neuron models to experimental data. Physiol Rev.

[36] Vlachos A, Ikenberg B, Lenz M, Becker D, Reifenberg K, Bas-orth C, Deller T (2013). Synaptopodin regulates denervation-induced homeostatic synaptic plasticity. PNAS.

[37] Rall W, Rinzel J (1973). Branch input resistance and steady attenuation for input to one branch of a dendritic neuron model. Biophys J.

[38] Cazé RD, Humphries M, Gutkin BS (2013). Passive Dendrites Enable Single Neurons to Compute Linearly Non-separable Functions. PLoS Comput Biol.

[39] Rall W, Reiss RF (1964). Theoretical significance of dendritic trees for neuronal input-output relations. Neural Theory Model.

[40] Koch C, Poggio TA, Torre V (1983). Nonlinear interactions in a dendritic tree: localization, timing, and role in information processing. PNAS.

[41] Branco T, Häusser M (2011). Synaptic integration gradients in single cortical pyramidal cell dendrites. Neuron.

[42] Branco T, Clark BA, Häusser M (2010). Dendritic discrimination of temporal input sequences in cortical neurons. Science.

[43] Poirazi P, Brannon T, Mel BW (2003). Pyramidal neuron as two-layer neural network. Neuron.

[44] Poirazi P, Brannon T, Mel BW (2003). Arithmetic of subthreshold synaptic summation in a model CA1 pyramidal cell. Neuron.

[45] Gidon A, Segev I (2012). Principles Governing the Operation of Synaptic Inhibition in Dendrites. Neuron.

[46] Cuntz H, Mathy A, Häusser M (2012). A scaling law derived from optimal dendritic wiring. PNAS.

[47] Carnevale NT, Tsai KY, Claiborne BJ, Brown TH (1997). Comparative electrotonic analysis of three classes of rat hippocampal neurons. J Neurophysiol.

[48] Carnevale NT, Johnston D (1982). Electrophysiological characterization of remote chemical synapses. J Neurophysiol.

[49] Holmes WR, De Schutter E (2009). Passive cable modeling. Comput. Model. Methods Neurosci.

[50] Waters J, Schaefer A, Sakmann B (2005). Backpropagating action potentials in neurones: measurement, mechanisms and potential functions. Prog Biophys Mol Biol.

[51] Caporale N, Dan Y (2008). Spike timing-dependent plasticity: a Hebbian learning rule. Annu Rev Neurosci.

[52] Sjöström PJ, Rancz EA, Roth A, Häusser M (2008). Dendritic excitability and synaptic plasticity. Physiol Rev.

[53] Lisman J, Spruston N (2010). Questions about STDP as a General Model of Synaptic Plasticity. Front Synaptic Neurosci.

[54] Reeves TM, Steward O (1988). Changes in the firing properties of neurons in the dentate gyrus with denervation and reinnervation: implications for behavioral recovery. Exp Neurol.

[55] Deller T, Orth CB, Vlachos A, Merten T, Del Turco D, Dehn D, Mundel P, Frotscher M (2006). Plasticity of synaptopodin and the spine apparatus organelle in the rat fascia dentata following entorhinal cortex lesion. J Comp Neurol.

[56] Donohue DE, Ascoli GA (2008). A comparative computer simulation of dendritic morphology. PLoS Comput Biol.

[57] Takasaki C, Okada R, Mitani A, Fukaya M, Yamasaki M, Fujihara Y, Shirakawa T, Tanaka K, Watanabe M (2008). Glutamate transporters regulate lesion-induced plasticity in the developing somatosensory cortex. J Neurosci.

[58] Butz M, Wörgötter F, Van Ooyen A (2009). Activity-dependent structural plasticity. Brain Res Rev.

[59] Overman JJ, Clarkson AN, Wanner IB, Overman WT, Eckstein I, Maguire JL, Dinov ID, Toga AW, Carmichael ST (2012). A role for ephrin-A5 in axonal sprouting, recovery, and activity-dependent plasticity after stroke. PNAS.

[60] Blennow K, Hardy J, Zetterberg H (2012). The neuropathology and neurobiology of traumatic brain injury. Neuron.

[61] Phinney AL, Deller T, Stalder M, Calhoun ME, Frotscher M, Sommer B, Staufenbiel M, Jucker M (1999). Cerebral amyloid induces aberrant axonal sprouting and ectopic terminal formation in amyloid precursor protein transgenic mice. J Neurosci.

[62] Šišková Z, Justus D, Kaneko H, Friedrichs D, Henneberg N, Beutel T, Pitsch J, Schoch S, Becker A, von der Kammer H, Remy S (2014). Dendritic Structural Degeneration Is Functionally Linked to Cellular Hyperexcitability in a Mouse Model of Alzheimer’s Disease. Neuron.

[63] Keck T, Mrsic-Flogel TD, Vaz Afonso M, Eysel UT, Bonhoeffer T, Hübener M (2008). Massive restructuring of neuronal circuits during functional reorganization of adult visual cortex. Nat Neurosci.

[64] Zepeda A, Aguilar-Arredondo A, Michel G, Ramos-Languren LE, Escobar ML, Arias C (2013). Functional recovery of the dentate gyrus after a focal lesion is accompanied by structural reorganization in the adult rat. Brain Struct Funct.

[65] Emoto K (2012). Signaling mechanisms that coordinate the development and maintenance of dendritic fields. Curr Opin Neurobiol.

[66] Scheff SW, Bernardo LS, Cotman CW (1978). Decrease in adrenergic axon sprouting in the senescent rat. Science.

[67] Gall C, Lynch G (1981). Fiber architecture of the dentate gyrus following ablation of the entorhinal cortex in rats of different ages: Evidence for two forms of axon sprouting in the immature brain. Neuroscience.

[68] Sousounis K, Baddour JA, Tsonis PA (2014). Aging and regeneration in vertebrates. Curr Top Dev Biol.

[69] Cuntz H, Forstner F, Borst A, Häusser M (2011). The TREES toolbox--probing the basis of axonal and dendritic branching. Neuroinformatics.

[70] Carnevale NT, Hines ML (2004). The NEURON Book.

[71] Aradi I, Holmes WR (1999). Role of multiple calcium and calcium-dependent conductances in regulation of hippocampal dentate granule cell excitability. J Comput Neurosci.

[72] Santhakumar V, Aradi I, Soltesz I (2005). Role of mossy fiber sprouting and mossy cell loss in hyperexcitability: a network model of the dentate gyrus incorporating cell types and axonal topography. J Neurophysiol.

[73] Chiang P-H, Wu P-Y, Kuo T-W, Liu Y-C, Chan C-F, Chien T-C, Cheng J-K, Huang Y-Y, Chiu C-D, Lien C-C (2012). GABA Is Depolarizing in Hippocampal Dentate Granule Cells of the Adolescent and Adult Rats. J Neurosci.

[74] Hines ML, Morse T, Migliore M, Carnevale NT, Shepherd GM (2004). ModelDB: A Database to Support Computational Neuroscience. J Comput Neurosci.

[75] Li X, Ascoli GA (2006). Computational simulation of the input-output relationship in hippocampal pyramidal cells. J Comput Neurosci.

