# Supplementary material for: A general homeostatic principle following lesion induced dendritic remodeling
Source: Acta Neuropathol Commun. 2016 Feb 25;4:19. doi: 10.1186/s40478-016-0285-8 (PMC4766619; doi:10.1186/s40478-016-0285-8)
Supplement: Supplementary file 1 — Supplementary Material. (PDF 580 kb) [file 40478_2016_285_MOESM1_ESM.pdf]

Supplementary Information

A general homeostatic principle following lesion induced dendritic remodeling

Steffen Platschek, Hermann Cuntz, Mario Vuksic, Thomas Deller, Peter Jedlicka

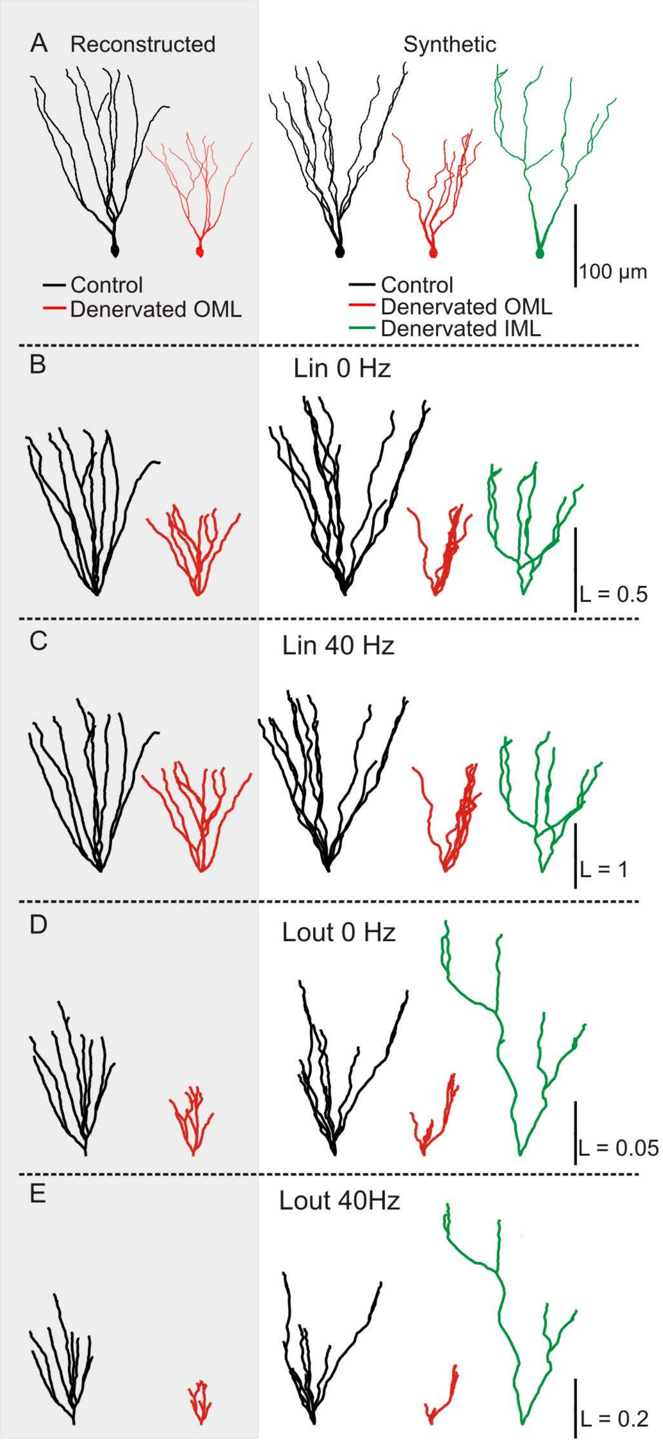
A
Reconstructed
Synthetic
100 μm
Control
Denervated OML
Control
Denervated OML
Denervated IML
B
Lin 0 Hz
L = 0.5
C
Lin 40 Hz
L = 1
D
Lout 0 Hz
L = 0.05
E
Lout 40Hz
L = 0.2

Figure S1

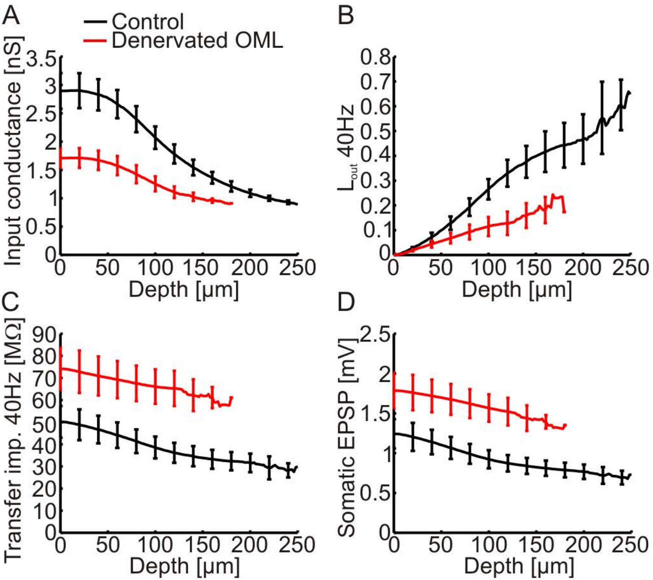
A
Control
Denervated OML
Input conductance [nS]
Depth [μm]
B
L_{out} 40Hz
Depth [μm]
C
Transfer imp. 40Hz [MΩ]
Depth [μm]
D
Somatic EPSP [mV]
Depth [μm]

Figure S2

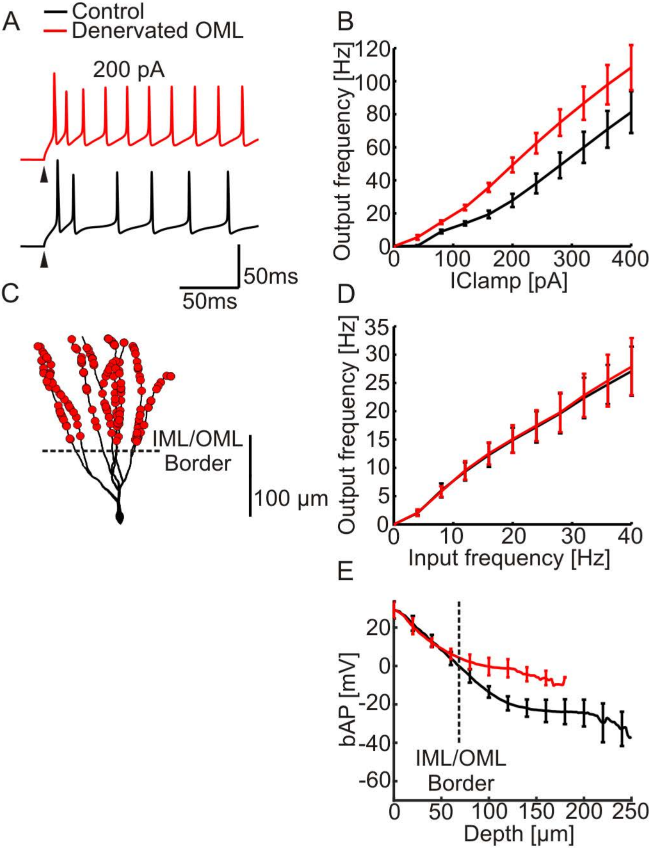
A
Control
Denervated OML
200 pA
50ms
50ms
B
Output frequency [Hz]
120
100
80
60
40
20
0
0
100
200
300
400
IClamp [pA]
C
IML/OML
Border
100 µm
D
Output frequency [Hz]
35
30
25
20
15
10
5
0
0
10
20
30
40
Input frequency [Hz]
E
bAP [mV]
20
0
-20
-40
-60
IML/OML
Border
0
50
100
150
200
250
Depth [µm]

Figure S3

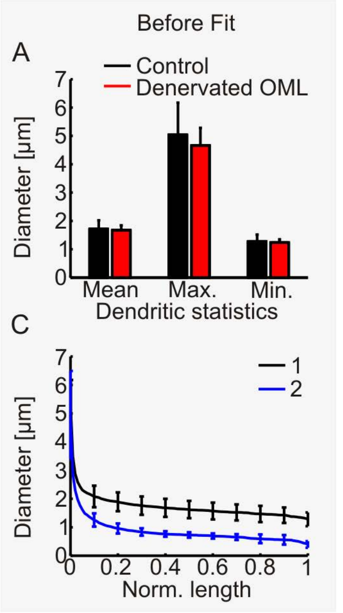

Before Fit
After Fit
A
B
C
D
Control
Denervated OML
Diameter [µm]
Mean
Max.
Min.
Dendritic statistics
1
2
Norm. length

Figure S4

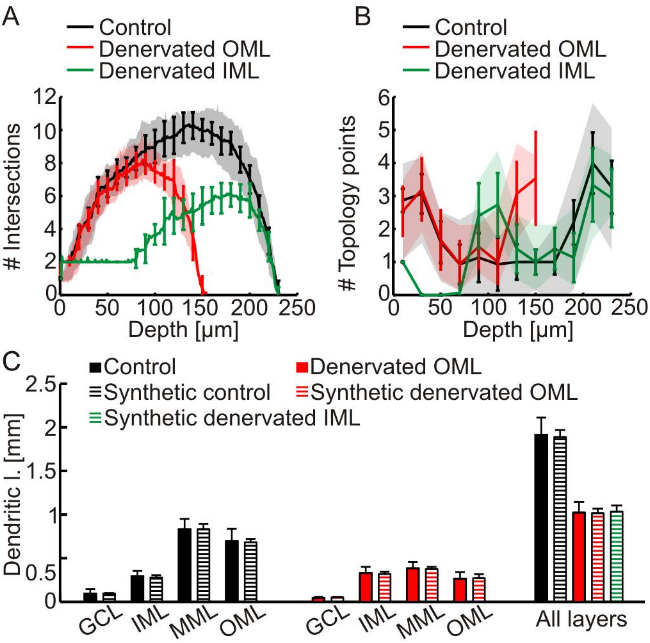
A
Control
Denervated OML
Denervated IML
Intersections
Depth [µm]
B
Control
Denervated OML
Denervated IML
Topology points
Depth [µm]
C
Control
Denervated OML
Synthetic control
Synthetic denervated OML
Synthetic denervated IML
Dendritic l. [mm]
GCL
IML
MML
OML
GCL
IML
MML
OML
All layers

Figure S5

Supplementary figure legends

Fig. S1. Morphoelectrotonic transforms of control and denervated granule cells.

To visualize the voltage attenuation in granule cell compartmental models, we computed morphoelectrotonic transforms (1) using the impedance tools built into NEURON (2). The morphoelectrotonic transformation consists of replacing metric lengths in the morphological structure with electrotonic length values while preserving their topology and local angles. In this case the anatomical length of each segment was replaced by the natural logarithm of the voltage attenuation along that segment. The distance between two points on the dendrite then provides a measure for the degree of their electrical coupling. The morphoelectrotonic transformations are shown for sample control (black) and denervated granule cells (red), and corresponding morphological models (right) of control (black), OML lesion (red) and IML lesion (green). (*A*) Original morphologies are shown. (*B* & *C*) Somatopetal (inward) voltage attenuation (L_{in}) for steady state current injection and for 40 Hz frequency respectively. (*D* & *E*) Somatofugal (outward) voltage attenuation (L_{out}) for steady state current injection and for 40 Hz frequency respectively. Both somatopetal and somatofugal voltage attenuation was decreased as compared to the control counterparts in all cases (n = 15), whether under steady state currents or fast currents corresponding to action potential speeds (40 Hz).

Fig. S2. Denervation-induced changes in local input conductance, somatodendritic voltage attenuation, transfer impedance, and EPSP attenuation in compartmental models of granule cells as a function of depth in the molecular layer.

(*A*) Local input conductance values for denervated (red) and control (black) granule cells (n = 15, each; same cells also in the following three panels). (*B*) Outward, somatodendritic voltage attenuation (L_{out}) resulting from the decay of a sinusoidal signal (40 Hz) applied to the soma. (*C*) Transfer impedance (ratio of remote voltage change vs. local current injection) at 40 Hz. (*D*) Attenuation of single EPSPs along the depth of the molecular layer.

Fig. S3. Denervation-induced changes of intrinsic excitability of model granule cells.

As a direct result of lower input conductances (Fig. S2*A*), the number of action potentials evoked during current stimulation in the soma *(A)* was greater in the denervated granule cell. (*A) Left:* Somatic membrane voltage traces in a control (black) and a denervated (red) model granule cell in response to +200 pA somatic current injections in the active AH-model (see Methods). Note an increase in the number of evoked action potentials in the denervated granule cell. Group results are depicted in (*B*). (*C*) Excitatory synapses were randomly distributed in the denervated layer (OML) using the same synaptic density in both groups. *(D)* Corresponding firing rates in response to increasing frequency of stochastic activation of dendritic synapses in the OML. Dendritic retraction leads to a decrease in number of synapses, given the unchanged synapse density (6). Note the precise adjustment of

the excitability enabling the lower number of synapses to efficiently drive the neuron. *(E)* Simulations of action potential backpropagation (bAP) in active compartmental models of control (black) and denervated (red) granule cells (n = 15, each) as a function of depth in the molecular layer. The border between inner and outer molecular layer (obtained from the reconstructed cell data) is indicated by a dashed line. Similarly to simulations in passive models (Fig. 1B), bAP amplitudes increased selectively in the denervated outer molecular layer (OML) but not in the inner molecular layer (IML).

Fig. S4. Realistic dendritic diameter taper in morphologies of dentate granule cells.

The diameter values in reconstructions from Vuksic et al. (6) were overestimated since they were obtained from fluorescent image stacks instead of biocytin fills. To adjust this, diameter values corresponding to a quadratic diameter taper (7, 8) were mapped onto the dendrites to obtain a diameter taper that both matched previous passive compartmental models of granule cells by Schmidt-Hieber et al. (9) and favored synaptic democracy (see Methods for details). (*A & B*) Comparison of dendritic diameter values in control and denervated granule cells before *(A)* and after *(B)* the quadratic taper fit. (*C & D*) Diameters of the entire dendritic tree were sorted and plotted against normalized total dendritic length to compare diameters in reconstructed granule cells with biocytin labeled granule cells from Schmidt-Hieber et al. before *(C)* and after *(D)* the quadratic taper fit. 1 (black): Vuksic et al. (6); 2 (dark blue): Schmidt-Hieber et al. (9).

Fig. S5. Calibration of synthetic dentate gyrus granule cells

(*A*) Sholl-like intersections (number of intersections of each tree with a plane at increasing somatodendritic depth) for original (reconstructed) and synthetic cells (continuous lines: synthetic cells; shaded areas represent the mean ± standard deviation of reconstructed data). (*B*) Histograms for the distribution of topological points (branch points and termination points) in original morphologies (control, denervated OML) and in synthetic morphologies (control, denervated OML, denervated IML). (*C*) Layer-specific dendritic length for original and synthetic morphologies.

References

1. Zador AM, Agmon-Snir H, Segev I (1995) The morphoelectrotonic transform: a graphical approach to dendritic function. *J Neurosci* 15:1669–1682.

2. Carnevale NT, Hines ML (2004) *The NEURON Book.* (Cambridge University Press).

3. Aradi I, Holmes WR (1999) Role of multiple calcium and calcium-dependent conductances in regulation of hippocampal dentate granule cell excitability. *J Comput Neurosci* 6:215–235.

4. Ferrante M, Migliore M, Ascoli GA (2009) Feed-forward inhibition as a buffer of the neuronal input-output relation. *PNAS* 106:18004–18009.

5. Schmidt-Hieber C, Bischofberger J (2010) Fast sodium channel gating supports localized and efficient axonal action potential initiation. *J Neurosci* 30:10233–10242.

6. Vuksic M et al. (2011) Unilateral entorhinal denervation leads to long-lasting dendritic alterations of mouse hippocampal granule cells. *Exp Neurol* 230:176–185.

7. Cuntz H, Borst A, Segev I (2007) Optimization principles of dendritic structure. *Theor Biol Med Model* 4:21.

8. Cuntz H, Forstner F, Borst A, Häusser M (2010) One rule to grow them all: a general theory of neuronal branching and its practical application. *PLoS Comput Biol* 6:e1000877.

9. Schmidt-Hieber C, Jonas P, Bischofberger J (2007) Subthreshold dendritic signal processing and coincidence detection in dentate gyrus granule cells. *J Neurosci* 27:8430–8441.
